# Account of Diseases in an Eastern District of London, from the 20th of April to the 20th of May

**Published:** 1799-06

**Authors:** 


### STATE OF DISEASES IN LONDON.

*Account of Diseases in an Eastern District of London, from the 20th of April to the 20th of May.*

| ACUTE DISEASES. | No. of Cases. |
|---|---|
| Typhus Mitior | 3 |
| Peripneumonia Notha | 4 |
| Catarrh | 7 |
| Ophthalmia | 5 |
| Acute Rheumatism | 7 |
| **CHRONIC DISEASES.** | |
| Dyspnœa | 10 |
| Cough | 12 |
| Cough and Dyspnœa | 14 |
| Phthisis Pulmonalis | 6 |
| Hæmoptoe | 4 |
| Ascites | 3 |
| Anasarca | 4 |
| Hydrothorax | 2 |
| Hepatitis Chronica | 3 |
| Cephalalgia | 10 |
| Vertigo | 2 |
| Hemicrania | 3 |
| Ophthalmia | 4 |
| Epistaxis | 2 |
| Amenorrhœa | 5 |
| Menorrhagia difficilis | 3 |
| Chlorosis | 4 |
| Enterodynia | 3 |
| Diarrhœa | 5 |
| Vomitus | 2 |
| Dyspepsia | 7 |
| Gastrodynia | 6 |
| Hæmorrhois | 2 |
| Dysuria | 4 |
| Herpes | 3 |
| Tinea | 2 |
| Psora | 1 |
| Jaundice | 2 |
| Hysteria | 4 |
| Hypochondriasis | 5 |
| Palpitation of the Heart | 1 |
| Gout | 1 |
| Chronic Rheumatism | 12 |
| **PUERPERAL DISEASES.** | |
| Ephemera | 3 |
| Menorrhagia lochialis | 3 |
| Mastrodynia | 5 |
| Rhagas Papillæ | 3 |
| **INFANTILE DISEASES.** | |
| Aphthæ | 2 |
| Herpetic Eruptions | 3 |
| Tinea | 4 |
| Tabes Mesenterica | 2 |
| Ophthalmia | 2 |
| Hooping Cough | 5 |

Although we have advanced beyond the period in which winterly diseases generally prevail, the weather still continuing very cold, we have had a large number of cases of catarrh, dyspnœa, cough, and rheumatism.

The instances of ophthalmia, which have lately occurred, have been uncommonly numerous. This may probably be attributed to the long continuance of east and north-east winds. This disease has been distinguished by nosologists, according to the part of the eye which has been the seat of it, or according to the remote causes by which it has been produced. It has sometimes its seat in the membranes of the eye, when it has been denominated ophthalmia membranarum: when it has appeared chiefly on the edges of the eye-lids, it has been termed ophthalmia tarsi.

These two kinds are, however, very frequently connected. This disease often appears in connection with scrophula or syphilis, in which case it is very liable to occur on every slight occasion.

It has lately been produced in several instances by exposure to a cold air, after which has been observed a fulness of the vessels of the tunica conconjunctiva, accompanied with considerable pain, an effusion of tears, and intolerance of light. There is generally a discharge of an acrimonious fluid, which during the night closes the eyes, and renders some emollient application necessary for the more easy separation of the eye-lids.

This disease, though the symptoms at the first appearance may be very similar, admits of a great variety of treatment, depending on the general state of the constitution, the original cause, and varying circumstances of the case. These have been so accurately distinguished, and so judicious a mode of treatment has been recommended by Mr. Ware, in his different writings on the subject, that we cannot do better than direct the attention of the practitioner to this source of information.

